# Distribution and diversity of olefins and olefin-biosynthesis genes in Gram-positive bacteria

**DOI:** 10.1186/s13068-020-01706-y

**Published:** 2020-04-15

**Authors:** Maximilian Surger, Angel Angelov, Wolfgang Liebl

**Affiliations:** Chair of Microbiology, Technical University of Munich, Emil-Ramann-Str. 4, 85354 Freising-Weihenstephan, Germany

**Keywords:** *Micrococcales*, *Micrococcus luteus*, *OleABCD*, Phylogenetics, Olefin diversity, OleA

## Abstract

**Background:**

The natural production of olefins (unsaturated aliphatic hydrocarbons) by certain bacterial genera represents an alternative and sustainable source of biofuels and lubricant components. The biochemical steps of olefin biosynthesis via the ole pathway encoded by *oleABCD* have been unraveled recently, and the occurrence of olefins has been reported for several Gram-negative and Gram-positive bacteria. However, the distribution and diversity of olefins among the Gram-positive bacteria has not been studied in detail.

**Results:**

We report the distribution of olefin synthesis gene clusters in the bacterial domain and focus on the olefin composition and the determinants of olefin production within the phylum of *Actinobacteria*. The olefin profiles of numerous genera of the *Micrococcales* order were analyzed by GC/MS. We describe for the first time olefin synthesis in representatives of the genera *Pseudarthrobacter*, *Paenarthrobacter*, *Glutamicibacter*, *Clavibacter*, *Rothia*, *Dermacoccus*, *Kytococcus*, *Curtobacterium*, and *Microbacterium*. By exchange of the native *ole* genes of *Micrococcus luteus* with the corresponding genes of actinobacteria producing different olefins, we demonstrate that the olefin composition can be manipulated with respect to chain length and isomer composition.

**Conclusions:**

This study provides a catalogue of the diversity of olefin structures found in the *Actinobacteria.* Our *ole* gene swapping data indicate that the olefin structures are fundamentally determined by the substrate specificity of OleA, and at the same time by the availability of a sufficient supply of suitable fatty acyl-CoA substrates from cellular fatty acid metabolism. This makes OleA of Gram-positive bacteria a promising target for structural analysis and protein engineering aiming to generate olefin chain lengths and isomer profiles which are designed to match the requirements of various industrial applications.

## Background

**Fig. 1:**
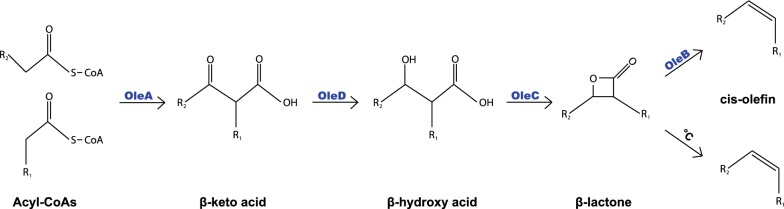
Olefin biosynthesis via the ole pathway according to Christenson et al. [6]. The thiolase OleA converts two acyl-CoAs in a non-decarboxylating “head-to-head” Claisen condensation to a β-keto acid. In a NADPH-dependent reaction, the dehydrogenase OleD reduces the β-keto acid to the corresponding β-hydroxy acid. The β-lactone synthetase OleC converts the β-hydroxy acid into a heat-unstable β-lactone. Finally, OleB decarboxylates the β-lactone to the cis olefin

Owing to the limited resources for fossil fuels and the ethical conflict over plant oil-derived fuels competing with food agriculture, synthesis of hydrocarbons in metabolically engineered microorganisms has become a sustainable alternative (Pfleger et al. [11]). The current routes for synthesis of diverse microbial hydrocarbons are largely based on fatty acid metabolism, and the enzymatic steps of the ole pathway (olefin synthesis pathway encoded by the operon *oleABCD*, consisting of the genes *oleA*, *oleBC*, and *oleD*) have been completely elucidated within the last few years. In the first reaction of this pathway (Fig. 1), catalyzed by OleA (protein encoded by *oleA*), two activated fatty acids undergo a non-decarboxylative head-to-head Claisen condensation that results in a β-keto acid. This intermediate acid is transferred without temporary release to the OleBCD complex, where it is reduced to a β-hydroxy acid by the NADH-dependent dehydrogenase OleD. The lactone synthase OleC forms a heat-labile internal ester between the β-hydroxy and the carboxyl group and one molecule of water is released. Finally, the lactone decarboxylase OleB liberates one molecule of CO_2_ and converts the internal lactone into an alkene with a cis-configured internal double bond (internal olefins) at the connection point of the two original fatty acid precursors (Beller et al. [3]) (Sukovich et al. [14]) (Frias et al. [7]) (Christenson et al. [4]) (Christenson, Richman et al. [6]) (Christenson, Jensen et al. [5]).

The nature of such internal olefin hydrocarbons was first characterized in the Gram-positive *Sarcina lutea* (now *Kocuria rhizophila*) (Albro and Dittmer [1]) (Tornabene et al. [17]), and the *oleABCD* genes were first described in the Gram-positive *Micrococcus luteus* NCTC2665 (Beller et al. [3]). Nevertheless, the catalytic mechanism of OleA as well as the order and nature of the reaction steps of the OleABCD enzymes, as summarized above, were finally elucidated mainly in the Gram-negative species *Shewanella oneidensis* (Sukovich et al. [14]) and *Xanthomonas campestris* (Frias et al. [7]) (Christenson et al. [4]) (Christenson et al. [6]) (Christenson et al. [5]). In addition, the first report on the phylogenetic distribution and diversity of olefin production in bacteria by Sukovich et al. [13] described bacterial olefin production as a predominately Gram-negative capability using species from four Gram-negative phyla (*Proteobacteria*, *Planctomycetes*, *Chloroflexi*, *Verrucomicrobia*) and one Gram-positive phylum (*Actinobacteria*) (Sukovich et al. [13]).

Regarding the potential application of such hydrocarbons as substitute fuels, it must be noted that a perfect substitute should be chemically identical to the existing fossil fuels, which are straight-chain and *iso*-branched C8–18 alkenes and alkanes. The spectrum of olefins from Gram-negative bacteria consists due to a predominantly straight-chain unsaturated fatty acid synthesis of C28–C31 monoalkenes, dienes, trienes and especially nonaenes, while Gram-positive bacteria due to their predominant branched chain fatty acid synthesis produce a spectrum of *iso*-branched, *anteiso*-branched, and straight-chain C20–30 monoalkenes (Sukovich et al., [13]). The fatty acid synthesis of Gram-positive bacteria is based primarily on the elongation of branched chain acyl-CoA primers, which is in contrast to Gram-negative bacteria where acetyl-CoA is exclusively used as a primer molecule. Those branched chain primers are provided by the branched chain amino acid catabolism, whose key enzymes have recently been described for the high-GC (high guanine plus cytosine content of the genome) Gram-positive *Micrococcus luteus* (Surger et al. [16]). Thus, olefins from Gram-positive bacteria by nature and without additional metabolic or protein engineering more closely resemble current fossil fuels than those from Gram-negative bacteria. Existing cracking technology could be applied to reduce the chain length of the Gram-positive olefins to make them better suited as drop-in fuels, i.e., compounds that can be added to existing fuels without further modification. On the other hand, also the native medium-chain olefins may be exploited as valuable constituents of lubricant formulations if they can be produced efficiently in high yields at economically viable costs.

This report provides an extensive survey about the great diversity of olefins produced by Gram-positive bacteria and creates a catalogue of Gram-positive olefin producers, making their OleABCD biosynthetic enzymes accessible for future biotechnological applications. Within the phylum *Actinobacteria*, we provide a detailed insight into the diversity of the fatty acid and olefin profiles of several strains per Gram-positive genus. Furthermore, we used *oleABCD* cluster and *oleA* exchange mutants to investigate to which extent the diversity of actinobacterial olefin profiles is determined by the substrate specificity of the OleABCD enzymes.

## Results

### Distribution of *oleABCD* genes within the bacterial domain

**Fig. 2:**
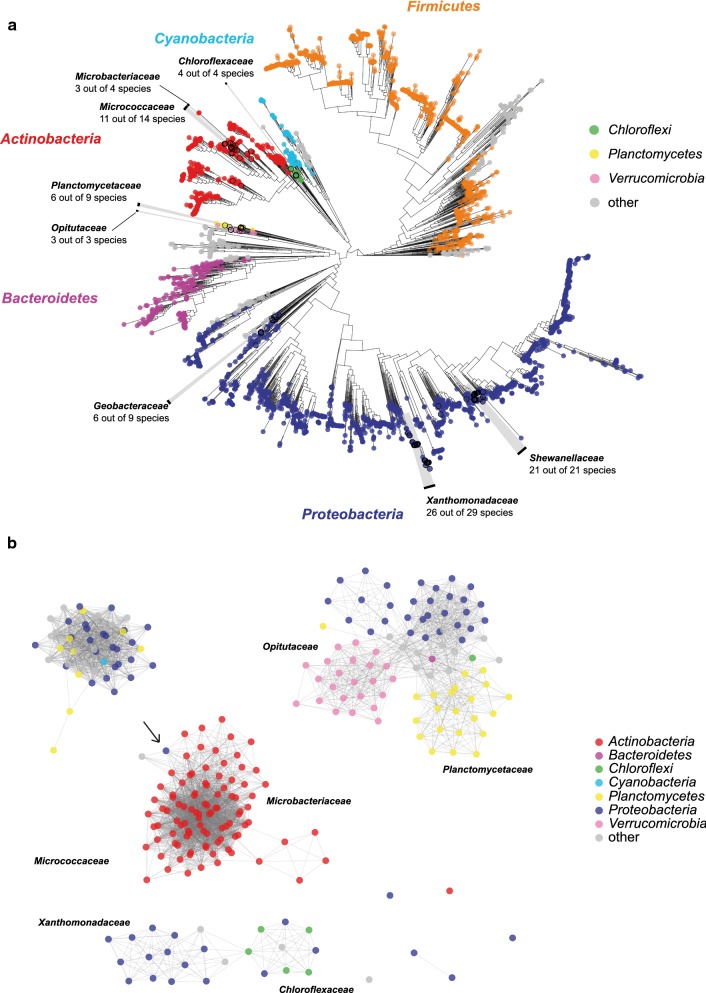
Occurrence of *ole* gene clusters in the bacterial tree of life. **a** Species containing *ole* gene clusters are marked with a black circle. The phylogenetic tree is adapted from [dataset] Segata et al. [12] and contains 3530 bacterial species. For selected families, the number of *ole* gene-containing species out of all the species in the tree is shown (black text). Similarity network analysis of OleA protein sequences between members of the same and all other *ole* gene cluster-possessing phyla. **b** A horizontal gene transfer event is marked using a black arrow

Using a combination of PROSITE pattern search and analysis of the gene neighborhood of potential OleA hits, we could detect *oleABCD* gene clusters in 1082 bacterial genomes, including incomplete genomes. No putative *ole* clusters could be detected in Archaea or Eukarya, as well as in some bacterial phyla (e.g., *Firmicutes* and *Bacteroidetes*). Mapping the *ole* cluster-containing genomes on the phylogenetic tree of Segata et al. [12] (underlying data available on https://github.com/angelovangel/oleA-distribution-paper-dataset) revealed that the *ole* gene-containing species are found in very diverse phylogenetic groups, forming in total seven clusters (three in the *Proteobacteria*, two in the *Actinobacteria* and one each in the *Chloroflexi*, *Planctomycetes* and *Verrucomicrobia*) (Fig. 2a). Notably, the distribution pattern of *ole* gene clusters was almost always ubiquitous at the genus rank level (i.e., all the members of a phylogenetic genus either did or did not possess *ole* gene clusters). Using similarity network analysis of OleA protein sequences, it was found that the OleA protein sequences in each phylum were more similar to each other than to proteins from the other phyla (Fig. 2b). This observation suggests an ancient origin of the protein, in general. Nevertheless, the clustering of OleA from the *Proteobacteria* species *Pseudohaleia rubra* with the OleAs from the *Actinobacteria* was observed too, which indicates that rare lateral gene transfer events have occurred.

### Diversity of olefin and fatty acid profiles in the *Micrococcales* order

**Fig. 3:**
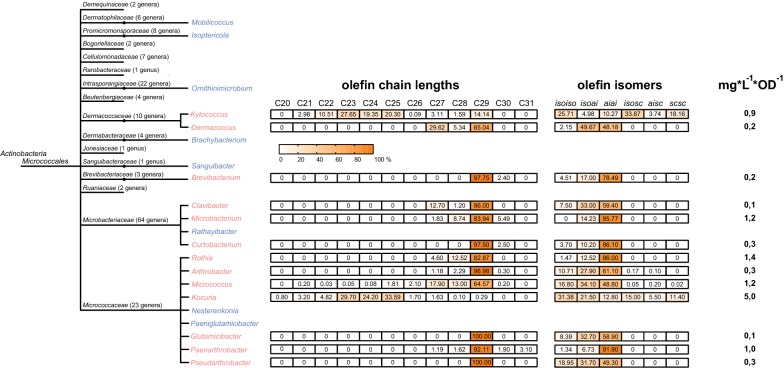
Summary of actinobacterial olefin producing genera. A simplified tree of the Gram-positive order *Micrococcales* within the *Actinobacteria* phylum is shown on the left. Genera are marked in red if they include species and strains from which olefins have been successfully extracted. Genera with only a positive in silico screening result for *oleABCD* are marked in blue. On the right, a representative average of the olefin profiles (chain lengths and isomers) of the olefin-synthesizing genera is shown, as well as the produced amounts. Abbreviations: *isoiso*, *iso*-branched at both ends; *isoai*, *iso*-branched at one end and *anteiso*-branched at the other end; *aiai*, *anteiso*-branched at both ends; *isosc*, *iso*-branched at one end and no branching at the other end; *aisc*, *anteiso*-branched at one end and no branching at the other end; *scsc*, no branching at both ends [15]

Within the phylum *Actinobacteria*, the *oleABCD* gene cluster-possessing genera were mostly found in the order *Micrococcales*. In order to expand the available structural and phylogenetic information about the olefins within this bacterial order, we acquired and analyzed 65 strains, belonging to the genera *Micrococcus*, *Kocuria*, *Arthrobacter*, *Brevibacterium/Curtobacterium* (homotypic synonyms), *Glutamicibacter*, *Clavibacter*, *Dermacoccus*, *Kytococcus*, *Microbacterium*, *Paenarthrobacter*, *Pseudarthrobacter*, and *Rothia*, for their potential olefin production capacity. The strains were grown in a complex medium at 30 °C to stationary phase and olefins and total fatty acids for each isolate were extracted and analyzed by GC/MS (gas chromatography/mass spectrometry). An overview of the olefin profiles and amounts produced by the investigated genera can be seen in Fig. 3, which shows a representative average of all isolates that could be assigned to the respective genera. It is noteworthy that the total amounts of olefins produced (normalized to optical densities of the cultures) revealed considerable variation between different strains belonging to the same genus, but the chain length and isomer profiles were usually similar within each genus. Additional files 1, 2, 3, 4, 5: Figures S1–S5 provide detailed reports on the olefin and fatty acid profiles of all single strains. Assuming a constant ratio of cell numbers per optical density and taking the comparable fatty acid levels for all *Micrococcales* strains into account, the total amounts produced by *Kocuria* strains were remarkable. In contrast, *Micrococcus*, *Microbacterium*, *Kytococcus*, *Paenarthrobacter*, and *Rothia* strains produced, on average, five times less than *Kocuria* strains, and *Arthrobacter*, *Pseudarthrobacter*, *Brevibacterium/Curtobacterium*, *Clavibacter*, and *Dermacoccus* strains produced only traces of olefins. The average olefin chain lengths in the order *Micrococcales* ranged from 27 to 29 carbon atoms, with the latter being the dominant length. The average olefin isomer profile, as expected for Gram-positive bacteria with predominant branched chain fatty acid synthesis, consisted exclusively of *iso*- and *anteiso (ai)*-branched carbon chains. The isomers were dominated by anteisoanteiso (*aiai*)-branched olefins. The genus *Kocuria* was again an exception. The chain lengths produced in this genus ranged from 21 to 27 carbon atoms, which means that the olefins were considerably shorter than the average *Micrococcales* olefins. Strain *Ky. sedentarius* TW93 also revealed an unusual *Micrococcales* olefin profile. It produced chain lengths of 21 to 25 carbons, similar to those from *Kocuria* strains, but also of 27 to 29 carbon atoms, similar to the average length produced by *Micrococcales* strains. In addition, *Kocuria* and *Kytococcus* strains showed notably greater shares of *isoiso*-branched (*iso*-branched at both ends) olefins and were able to produce also straight-chain olefins. A common spectrum of 14 to 17 carbon atoms long, especially *ai*-branched and saturated fatty acids, was observed in all *Micrococcales* strains. The fatty acid spectrum of the strain *Dermacoccus* sp. blue KL114 was an exception, with a shifted chain length ranging from 15 to 18 carbon atoms, especially *iso*-branched fatty acids and greater shares of unsaturated fatty acids. The spectrum of *Microbacterium imperiale* was special because of the greater shares of cyclic fatty acids (Additional file 5: Figure S5).

### Substrate specificity of OleA determines olefin profiles

**Fig. 4:**
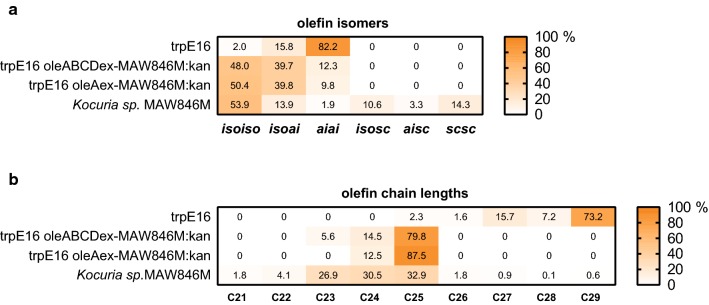
Olefin chain lengths and isomer distribution (in %) of *oleA* and *oleABCD* cluster exchange mutants of *Micrococcus luteus* trpE16 in complex medium compared to that from the parental strain and the *Kocuria sp.* MAW846M donor strain. The values are the mean of at least two biological replicates. The standard deviations of heat map values did not exceed 12%. Abbreviations: see Fig. 3

Despite sharing similar fatty acid spectra, there were obvious differences in the olefin profiles among the *Kocuria*, *Kytococcus*, and the rest of the *Micrococcales* strains. In order to study the underlying reasons for these differences, we generated recombinant strains in which the native *ole* genes or *ole* gene clusters of the *M. luteus* trpE16 strain were replaced by foreign gene or gene cluster homologs. In those strains, the native *M. luteus oleABCD* or *oleA* genes were replaced with the corresponding genes from *Kocuria*, *Kytococcus*, or other *Micrococcus* strains. In olefin synthesis, the initial condensation reaction between two fatty acid substrates is catalyzed by the condensing thiolase OleA. Thus, it can be expected that the substrate specificity of olefin synthesis is mainly determined by the affinity of OleA for specific substrates. To account for contributions by the other genes of the *ole* cluster, the effect of the exchange of the entire *oleABCD* cluster was compared to the exchange of *oleA* alone. In order to study the effect of transferring *ole* genes from *Kocuria* sp. MAW846 (donor) to *M. luteus* (acceptor), four strains were generated and grown in complex medium to stationary phase, before analysis of their olefin profiles (Fig. 4). The *Kocuria* donor strain produced *iso*-branched as well as straight-chain olefin isomers with chain lengths of 23 to 25 carbon atoms while the *M. luteus* parental strain naturally formed the general *Micrococcales ai*-branched isomer-dominated profile of mainly olefins with chains 29 carbon atoms long. The transfer of the *Kocuria oleABCD* cluster into *Micrococcus*, as well as *oleA* alone, led to the same strong shift from *ai*-branched to *iso*-branched olefin isomers; *M. luteus* still did not produce straight-chain olefins. In addition, the transfers led to a shift in olefin chain length from 29 to 25 carbons atoms in both cases and to the same extent.

**Fig. 5:**
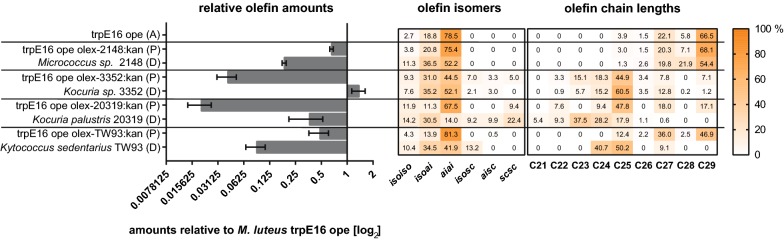
Relative olefin amounts (left panel), isomers, and chain lengths (right panel) of *oleA* exchange mutants (marked as P) of *Micrococcus luteus* ope in complex medium compared to that of the parental acceptor strain ope (marked as A) and to the respective *oleA* donor strain (marked as D). The amounts are expressed relative to those of the parental strain ope. The values shown are the mean of at least two biological replicates. Error bars represent standard deviation. Standard deviations of the heat map values did not exceed 10%. Abbreviations: see Fig. 3 [15]

We investigated additional *M. luteus oleA* exchange mutants, using *oleA* genes from the strains *Ko. palustris* DSM 20319, *Kocuria* sp. 3352, *Ky. sedentarius* TW93, and *Micrococcus* sp. 2148 (series of *oleA*ex strains, see Table 1). After growth in complex medium to stationary phase, the olefin profiles of these *oleA* exchange strains were again compared to the profiles of the respective donor and acceptor strains (Fig. 5). Olefin production in strain *Micrococcus* sp. 2148 was not as strictly dominated by olefins that were *ai*-branched and 29 carbon atoms long as is the case in *M. luteus* ope (ole promoter exchange strain in which the native *ole* promoter was replaced by the strong succinate dehydrogenase promoter). Nevertheless, if the native *oleA* in *M. luteus* ope was replaced by the gene from *Micrococcus* sp. 2148, the produced olefin profile was not changed. The resulting recombinant *M. luteus* exchange strain produced smaller amounts than the *M. luteus* parental strain; however, the new OleA enzyme produced more products than in its original metabolic background in *Micrococcus* sp. 2148. The olefin profile of *Kocuria sp.* 3352 was dominated by *ai*-branched olefins, but not to the same extent as found in the *M. luteus* ope strain. The chain lengths of the olefins in *Kocuria sp.* 3352 ranged from 23 to 27 carbon atoms with an excess of 25 carbon atoms, which is four carbon atoms shorter than the dominant chain length in *M. luteus* (C29, 29 carbon atoms). Replacement of the native *oleA* in *M. luteus* ope by the corresponding gene from *Kocuria sp.* 3352 allowed *M. luteus* to produce straight-chain olefins in complex medium and reduced the dominance of *ai*-branched isomers for the benefit of *iso*-branched isomers in *M. luteus*. In addition, the olefin chain length was shifted from mainly 29 carbon atoms to 25 carbon atoms. The switch in *oleA* was accompanied by a strong loss in the productivity of olefin synthesis compared to the parental strain. *Ko. palustris* DSM 20319 produced straight-chain, *iso*- and *ai*-branched olefin isomers. The length of the produced chains ranged from 21 to 25 carbon atoms with most olefins being 23 carbon atoms long. The transfer of the *Ko. palustris* DSM 20319 *oleA* gene to *M. luteus* ope allowed *M. luteus* to produce straight-chain olefins in complex medium, but the dominance of *ai*-branched olefin isomers remained. The range of chain lengths in *M. luteus* ope expressing the *Ko. palustris oleA* gene was expanded down to 22 carbon atoms, with the majority of olefins being 25 carbon atoms long. Again, in this case the transfer of *oleA* was accompanied by a significant decrease in the number of olefins compared to those produced in *M. luteus* ope. The *Kytococcus* strain in complex medium produced a relatively high percentage (> 40%) of straight-chain (C24) olefins, but had an affinity for *ai*-branched chains. Predominantly chains of 24 and 25 carbon atoms length were synthesized. When *oleA* of *M. luteus* ope was replaced by the gene from *Ky.* *sedentarius* TW93, the olefin profile of *M. luteus* shifted slightly toward shorter chains, but was still dominated by C29 olefins. The spectrum of olefin isomers of this *oleA* exchange strain was not changed compared to that in the *M. luteus* ope parental strain. The *M. luteus* exchange strain produced less olefins than the *M. luteus* parental strain, but the new OleA enzyme was much more productive in this strain than in its original metabolic background in *Ky.* *sedentarius* TW93.

**Table 1:** *Micrococcus luteus* trpE16 strains used in this study

Strain	Genotype and relevant phenotype	Source
trpE16	trpE16, Trp^−^ mutant of ATCC 27141	Kloos and Rose [10]
trpE16 *oleA*ex-MAW846M:kan	trpE16 with exchange of native *oleA* gene (Mlut_13230) against heterologous *oleA* gene from *Kocuria sp.* MAW846M; Kan^r^	This study
trpE16 *oleABCD*ex-MAW846M:kan	trpE16 with exchange of native *oleABCD* gene cluster (Mlut_13230) against heterologous *oleABCD* gene cluster from *Kocuria sp.* MAW846M; Kan^r^	This study
ope	trpE16 P_up_13230 (replacement of native *oleABCD* gene cluster promoter by strong succinate dehydrogenase promoter)	Surger et al. [16]
ope *oleA*ex-2148:kan	ope with exchange of native *oleA* gene (Mlut_13230) against heterologous *oleA* gene from *Micrococcus luteus* 2148; Kan^r^	This study
ope *oleA*ex-20319:kan	ope with exchange of native *oleA* gene (Mlut_13230) against heterologous *oleA* gene from *Ko. palustris* DSM 20319; Kan^r^	This study
ope *oleA*ex-3352:kan	ope with exchange of native *oleA* gene (Mlut_13230) against heterologous *oleA* gene from *Kocuria sp.* 3352; Kan^r^	This study
ope *oleA*ex-TW93:kan	ope with exchange of native *oleA* gene (Mlut_13230) against heterologous *oleA* gene from *Ky. sedentarius* TW93; Kan^r^	This study

*ope* ole promoter exchange, *Kan*^*r*^ kanamycin resistant

## Discussion

### Distribution of *oleABCD* genes in the bacterial domain, and diversity of olefin and fatty acid profiles in the *Micrococcales* order

By analysis of complete and incomplete genome sequence data, the *oleABCD* genes could be detected in certain lineages from five bacterial phyla, i.e., the *Proteobacteria* (*Gamma*- and *Deltaproteobacteria*), *Planctomycetes, Verrucomicrobia, Chloroflexi* and *Actinobacteria*, with apparently only rare occasions of lateral transfer having occurred. The order *Micrococcales* within the *Actinobacteria* phylum was the only Gram-positive order that revealed *oleABCD* cluster-possessing genera. Notably, we could find *ole* gene clusters in almost all of the species with complete genomes in the *Micrococcaceae* family. So far, olefin production from Gram-positive bacteria has been reported for single genera, such as *Micrococcus*, *Kocuria*, *Arthrobacter*, and *Brevibacterium* (Albro and Dittmer [1]) (Tornabene et al. [17]) (Frias et al. [8]) (Sukovich et al. [13]), but in each case only for single strains and without providing a broad picture of the distribution of olefin production. In this study, we systematically studied the olefin and fatty acids profiles from numerous strains (Additional file: 1, 2, 3, 4, 5 Figures S1–S5). We describe for the first time olefin synthesis for *Pseudarthrobacter*, *Paenarthrobacter*, *Glutamicibacter*, *Clavibacter*, *Rothia*, *Dermacoccus*, *Kytococcus*, *Curtobacterium*, and *Microbacterium*. The cultivation, extraction, and evaluation of in total 64 strains from 12 different genera (*Curtobacterium* is a homotypic synonym of *Brevibacterium*) of the order *Micrococcales* allowed both the documentation of fatty acid profiles and the identification of reference profiles for olefin production from various Gram-positive genera (Fig. 3). Most of the Gram-positive olefin producers exhibit profiles of particularly *anteiso*-branched olefins with chain lengths from 27 to 29 carbon atoms, with the latter being the dominant length. The *Kocuria* and *Kytococcus* strains represented exceptions and exhibited the capacity to synthesize olefins that are 21 to 27 carbon atoms long, which are unbranched, *iso*-branched, and *anteiso*-branched in equal amounts. In those cases, there appears to be either a selection of shorter *iso*-branched or unbranched fatty acid substrates by the biosynthetic enzymes (see below) and/or a greater supply of such shorter substrates in the cells. Olefin production was detected in all genera with available genome sequences containing *ole* gene orthologs for which lipid profiles of strains were analyzed in this study. Therefore, it is likely that also the other genera with sequenced genomes containing *ole* genes (marked blue in Fig. 3) are capable of olefin biosynthesis and produce olefins as components of their lipids.

### Substrate specificity of OleA that determines the olefin profile

The question to what extent the observed differences in the olefin profiles of *Kocuria* and *Kytococcus* strains as compared to *M. luteus* are determined by fatty acid metabolism and by the specificity of the OleABCD enzymes was addressed by swapping *ole* genes. After replacing the native *oleABCD* cluster as well as only the native *oleA* of *M. luteus* trpE16 with the corresponding genes from *Kocuria* strains (Figs. 3, 4), the olefin profile of the *M. luteus ole* exchange strains clearly mirrored the isomer and chain length distributions of the *Kocuria ole* gene donor strains. This indicates that the substrate specificity of OleA alone is the most important determinant of the olefin profile of the bacterial host. The supply of sufficient amounts of fatty acid substrates fitting the specificity of the strain’s OleA enzyme presumably represents another determining factor because the change of the olefin profile in *M. luteus* caused by introduction of the heterologous *Kocuria oleA* genes was accompanied by a drop of the produced olefin amounts (Fig. 4). Apparently, the full transition to the *Kocuria* olefin profiles in *M. luteus* was, in part, restricted by the lack of *iso*-branched and unbranched fatty acid chains, as well as of shorter fatty acid substrates within the *M. luteus* metabolic background (see top rows in Fig. 4a, b). The OleA enzyme of *K. palustris* DSM 20319 apparently has a relaxed isomer specificity. Upon introduction of this OleA enzyme into *M. luteus* an olefin profile dominated by *ai*-branched isomers was found (Fig. 5), determined by the predominance of *ai*-branched fatty acid primers in *M. luteus* (Surger et al. [16]). As a control, the exchange of the native *oleA* gene of *M. luteus* with another *Micrococcus oleA* from *Micrococcus* sp. 2148 was also performed. Although the encoded heterologous OleA amino acid sequence differed from *M. luteus* trpE16 OleA by 22%, far more than for the majority of the investigated *Micrococcus* strains in this study (97–99% OleA amino acid sequence identity), it displayed the same substrate specificity and by its expression the *M. luteus* trpE16 olefin profile was largely retained. Thus, it can be presumed that the OleA enzymes of all of the investigated *Micrococcus* strains have the same substrate specificity. Compared to the natural trpE16 ope acceptor strain a drop in the olefin amounts produced was observed though, which may be caused by less effective expression or reduced activity of the heterologous OleA enzyme.

*Ky. sedentarius* TW93 showed an olefin profile with greater shares of *iso*-branched and unbranched isomers, which was dominated by 24 to 25 carbon atom chains. Nevertheless, after transferring the corresponding *oleA* into the *M. luteus* trpE16 ope metabolic background, the olefin isomer and chain length profiles were very similar to the parental strain *M. luteus* trpE16 ope. This was accompanied in the new metabolic background by increased olefin amounts. Presumably the *Kytococcus* OleA enzyme, although it shares an amino acid sequence identity of only 47% with *M. luteus* trpE16 OleA, has a similar substrate specificity as the *M. luteus* OleA enzyme, but this substrate preference is not supported by the provided fatty acid substrates in the donor strain background. In *Ky. sedentarius* TW93, the main driver for the olefin isomer and chain length profile thus appears to be the fatty acid composition available in this host and not a very specific fatty acid preference of the OleA enzyme.

## Conclusions

According to the distribution of the *oleABCD* genes, olefin biosynthesis via the ole pathway is restricted to five bacterial phyla, and spreading of this trait by horizontal gene transfer appears to have occurred only rarely during evolution. Among the *Micrococcales* within the *Actinobacteria* phylum, the only Gram-positive order where the *ole* genes were found, most olefin producers exhibit profiles of particularly *anteiso*-branched olefins with chain lengths from 27 to 29 carbon atoms, with the exception of *Kocuria* and *Kytococcus* strains which synthesize shorter olefins of 21 to 27 carbon atoms length. Gene swapping experiments using *M. luteus* as a host organism demonstrate that the substrate specificity of OleA, besides the fatty acid composition available, is the key determinant for the olefin profile produced. These results can serve as a foundation for future research to engineer (i) the substrate specificity of this important class of enzymes and/or (ii) the fatty acyl-CoA precursor pool in the producer cells with the aim to construct strains tailored to produce specific olefins for industrial applications.

## Materials and methods

### Bacterial strains and growth conditions

A tryptophan auxotroph of *M. luteus* (formerly, *M. lysodeikticus*) ISU, trpE16 (Kloos and Rose [10]) (Angelov et al. [2]), and its derivatives (Table 1) were grown in lysogeny broth (LB) or modified Naylor medium at 30 °C. *M. luteus* trpE16 was used as a host for DNA manipulations and as a reference strain. Naylor medium had a pH of 7.2 and contained per liter 10 g sodium glutamate × H_2_O, 7 g glucose, 5 g NH_4_Cl, 2 g K_2_HPO_4_, 100 mg MgSO_4_ × 7H_2_O, 100 mg tryptophan, 10 mg biotin, 4 mg FeSO_4_ × 7H_2_O, and 2 mg MnCl_2_ × 4H_2_O and was prepared using ultrapure water. Where appropriate, the growth media were supplemented with 60 µg/mL kanamycin. All the other *Micrococcales* strains (Table 2) were grown in LB at 30 °C to stationary phase and used for olefin as well as total fatty acid extraction.

**Table 2:** Other *Micrococcales* strains used in this study

Strain	Database number and synonyms	Source
*Arthrobacter agilis*^a^	*Micrococcus agilis* (basonym)	TUM Chair of Microbiology strain collection (K. H. Schleifer, 614)
*Arthrobacter atrocyaneus*	LMG 3814, ATCC 13752	TUM Chair of Microbiology strain collection (K. H. Schleifer, 454)
*Arthrobacter aurescens*	DSM 20116, LMG 3815	TUM Chair of Microbiology strain collection (K. H. Schleifer, 84/487)
*Arthrobacter citreus*	LMG 16338, DSM 20133	TUM Chair of Microbiology strain collection (K. H. Schleifer, 447/256)
*Arthrobacter crystallopoietes*	LMG 3819, DSM 20117	TUM Chair of Microbiology strain collection (K. H. Schleifer, 85/440)
*Arthrobacter oxydans*	LMG 3816, DSM 20119	TUM Chair of Microbiology strain collection (K. H. Schleifer, 91/486)
*Arthrobacter polychromogenes*	LMG 16679, DSM 20136	TUM Chair of Microbiology strain collection (K. H. Schleifer, 522)
*Arthrobacter sp.*^a^	*Filibacter limicola* (designation in collection)	TUM Chair of Microbiology strain collection (K. H. Schleifer, 424)
*Arthrobacter sulfureus*	LMG 16694, DSM 20167	TUM Chair of Microbiology strain collection (K. H. Schleifer, 523)
*Arthrobacter uratoxydans*	LMG 16220, ATCC 21749	TUM Chair of Microbiology strain collection (K. H. Schleifer, 461)
*Brevibacterium casei*	ATCC 35513, NCDO 2048	TUM Chair of Microbiology strain collection (K. H. Schleifer, 491)
*Brevibacterium casei*	NCDO 2050, WS 2124	TUM Chair of Microbiology strain collection (K. H. Schleifer, 492)
*Brevibacterium sp.* Ap13	–	Fecal sample, Laguna Aparejos, Argentina, (J. Dib, PROIMI-CONICET)
*Clavibacter michiganensis* sp. *nebraskensis*	DSM 7483	DSMZ
*Curtobacterium albidum*	LMG 8759, *Brevibacterium albidum* (homotypic synonym)	TUM Chair of Microbiology strain collection (K. H. Schleifer, 465)
*Curtobacterium citreum*	LMG 8786, *Brevibacterium citreum* (homotypic synonym)	TUM Chair of Microbiology strain collection (K. H. Schleifer, 457)
*Curtobacterium flaccumfaciens pv. Betae*	LMG 3596, *Corynebacterium flaccumfaciens pv. Betae* (homotypic synonym)	TUM Chair of Microbiology strain collection (K. H. Schleifer, 485)
*Curtobacterium luteum*	LMG 8787, *Brevibacterium luteum* (homotypic synonym)	TUM Chair of Microbiology strain collection (K. H. Schleifer, 460)
*Curtobacterium pusillum*	LMG 8788, *Brevibacterium pusillum* (homotypic synonym)	TUM Chair of Microbiology strain collection (K. H. Schleifer, 449)
*Dermacoccus sp.* blue KL114	*M. or blue* (designation in collection)	TUM Chair of Microbiology strain collection (K. H. Schleifer, 606)
*Glutamicibacter nicotinae*	DSM 20123, ATCC 15236, *Arthrobacter nicotinae* (basonym)	TUM Chair of Microbiology strain collection (K. H. Schleifer, 451/86)
*Glutamicibacter protophormiae*	LMG 16324, DSM 20168, *Arthrobacter protophormiae* (basonym)	TUM Chair of Microbiology strain collection (K. H. Schleifer, 467)
*Ko. palustris* DSM 11925	CCM 4949, NBRC 16318	DSMZ
*Ko. palustris* DSM 20319^a^	DBM 385, *Micrococcus varians* (basonym)	DSMZ
*Ko. palustris* DSM 20320^a^	KH 113, *Micrococcus varians* (basonym)	DSMZ
*Kocuria rosea*	ATCC 186, NCTC 7523, *Micrococcus roseus* (basonym)	TUM Chair of Microbiology strain collection (W. Liebl)
*Kocuria rosea*^a^	ATCC 186, NCTC 7523, *Micrococcus roseus* (basonym)	TUM Chair of Microbiology strain collection (K. H. Schleifer, 615)
*Kocuria sp.* 3312^a^	–	Skin/nail/mucosal (M. Köberle TUM Department of Dermatology and Allergology)
*Kocuria sp.* 3352^a^	–	Skin/nail/mucosal (M. Köberle TUM Department of Dermatology and Allergology)
*Kocuria sp.* AT3343^a^	*M. luteus* AT3343 (designation in collection)	TUM Chair of Microbiology strain collection (W. Liebl)
*Kocuria sp.* MAW846M	*Micrococcus varians* (designation in collection)	TUM Chair of Microbiology strain collection (W. Liebl)
*Ky. sedentarius* TW93	ATCC 14392, DSM 20547, *Micrococcus sedentarius* (basonym)	TUM Chair of Microbiology strain collection (W. Liebl)
*Microbacterium imperiale*	WS 1959, *Brevibacterium imperiale* (basonym)	TUM Chair of Microbiology strain collection (K. H. Schleifer, 489)
*M. aurantiacus*	ATCC 11731	TUM Chair of Microbiology strain collection (W. Liebl)
*M. luteus* A31658	–	TUM Chair of Microbiology strain collection (W. Liebl)
*M. luteus* ATCC 15800	ML 53-40	TUM Chair of Microbiology strain collection (W. Liebl)
*M. luteus* ATCC 27141	JCM 3347	TUM Chair of Microbiology strain collection (W. Liebl)
*M. luteus* CCM 1339	–	TUM Chair of Microbiology strain collection (W. Liebl)
*M. luteus* DSM 20030	ATCC 4698, CCM 169	TUM Chair of Microbiology strain collection (W. Liebl)
*M. luteus* JW6	–	TUM Chair of Microbiology strain collection (W. Liebl/K. H. Schleifer, 600)
*M. luteus* MAW843	–	TUM Chair of Microbiology strain collection (W. Liebl)
*M. luteus* MAW845M	–	TUM Chair of Microbiology strain collection (W. Liebl)
*M. luteus* VM3	–	TUM Chair of Microbiology strain collection (W. Liebl)
*M. lylae* TW226	ATCC 27566, DSM 20315	TUM Chair of Microbiology strain collection (W. Liebl)
*Microbacterium imperiale*	WS 1959, *Brevibacterium imperiale* (basonym)	Skin/nail/mucosal (M. Köberle TUM Department of Dermatology and Allergology)
*Micrococcus sp.* 1306^a^	–	Skin/nail/mucosal (M. Köberle TUM Department of Dermatology and Allergology)
*Micrococcus sp.* 2105^a^		Skin/nail/mucosal (M. Köberle TUM Department of Dermatology and Allergology)
*Micrococcus sp.* 2148^a^	–	Skin/nail/mucosal (M. Köberle TUM Department of Dermatology and Allergology)
*Micrococcus sp*. 2181^a^	–	Skin/nail/mucosal (M. Köberle TUM Department of Dermatology and Allergology)
*Micrococcus sp.* 2228^a^	–	Skin/nail/mucosal (M. Köberle TUM Department of Dermatology and Allergology)
*Micrococcus sp.* 2525^a^	–	Skin/nail/mucosal (M. Köberle TUM Department of Dermatology and Allergology)
*Micrococcus sp.* 2559^a^	–	Skin/nail/mucosal (M. Köberle TUM Department of Dermatology and Allergology)
*Micrococcus sp.* 3185^a^	–	Laguna Diamante, Argentina (J. Dib, PROIMI-CONICET)
*Micrococcus sp.* 69	–	Laguna Diamante, Argentina (J. Dib, PROIMI-CONICET)
*Micrococcus sp.* 70	–	Water sample, Laguna Azul, Argentina (J. Dib, PROIMI-CONICET)
*Micrococcus sp.* A1	–	Water sample, Laguna Azul, Argentina (J. Dib, PROIMI-CONICET)
*Micrococcus sp.* A7	–	TUM Chair of Microbiology strain collection (W. Liebl)
*Micrococcus sp.* D12	–	Sediment sample, Laguna Diamante, Argentina (J. Dib, PROIMI-CONICET)
*Micrococcus sp*. H5	–	Water sample, Laguna Huaca Huasi, Argentina (J. Dib, PROIMI-CONICET)
*Micrococcus sp.* V7	–	Water sample, Laguna Vilama, Argentina (J. Dib, PROIMI-CONICET)
*Paenarthrobacter ilicis*	DSM 20138, LMG 3659, *Arthrobacter illicis, Corynebacterium ilicis* (basonym)	TUM Chair of Microbiology strain collection (K. H. Schleifer (469/90)
*Pseudarthrobacter chlorophenolicus*	DSM 12829	DSMZ
*Rothia sp.*^a^	DSM 20321, ATCC 27572, *Micrococcus kristinae* (designation in collection)	TUM Chair of Microbiology strain collection (W. Liebl)

*ATCC* American Type Culture Collection, *CCM* Czech Collection of Microorganisms, *DBM* Collection of Yeasts and Industrial Microorganisms, *DSMZ* German Collection of Microorganisms and Cell Cultures, *JCM* Japan Collection of Microorganisms, *LMG* Laboratorium voor Microbiologie en Microbiele Genetica, *NBRC* Biological Resource Center, National Institute of Technology and Evaluation (NITE), *NCDO* National Collection of Dairy Organisms (today incorporated into *NCIMB* National Collection of Industrial, Food and Marine Bacteria), *WS* Microorganism Collection Weihenstephan

^a^ For some strains, the strain designation was confirmed by 16S rDNA sequencing

### Production of the phylogenetic tree

The phylogenetic analysis for the presence/absence of *ole* gene clusters was performed using the following pipeline: putative OleA proteins were first obtained using the PROSITE search pattern EPxx[AS]x(14,18)DxxNACL against the nr database. Bacterial genomes with potential ole cluster were further verified by searching for *oleBCD* gene homologs in the close vicinity of the *oleA* gene (± 5000 bp, using *M. luteus* OleBC and OleD as queries and blastp cutoff *e* <= 10^−5^). Incomplete bacterial genomes with no OleA hits as well as OleA hits with no support for clustering with OleBCD were discarded. The list of complete bacterial genomes was obtained from Ensembl genomes release 38 (https://ensemblgenomes.org/pub/bacteria/release-38). The data about the presence of *ole* gene clusters was mapped on the phylogenetic tree of Segata et al. [12] which contains 3737 genomes (data available on https://github.com/angelovangel/oleA-distribution-paper-dataset). The phylogenetic tree was generated with the ggtree package in R (Yu et al. [18]).

### OleA sequence similarity network (SSN)

The similarity network analysis was performed using the EFI Enzyme Similarity server (https://efi.igb.illinois.edu/efi-est/, Gerlt et al. [9]). For this analysis, a total of 1349 putative OleA protein sequences were used, which resulted in 939 nodes in the SSN. The generated network used a similarity threshold of E < 10^−5^, i.e., proteins with this or higher level of amino acid sequence similarity are connected with edges. The length of the edges is proportional to the similarity score. Proteins with level of amino acid sequence identity higher than 75% were represented as one node.

### Construction of *M. luteus oleABCD* and *oleA* exchange (olex) mutants

Homologous regions of DNA sequences upstream and downstream of gene insertion sites, selection markers, and heterologous gene sequences were amplified by polymerase chain reaction (PCR) using Q5 polymerase (New England Biolabs). The Gibson assembly method (New England Biolabs) was used to assemble the PCR products with overlaps. The in vitro Gibson assembly reactions (~ 0.2–0.4 µg DNA) were added directly to *M. luteus* cells for uptake via natural transformation and plated on LB plates supplemented with the appropriate antibiotic, as described previously (Angelov et al. [2]). The correctness of the alterations in the *M. luteus* genome was confirmed by PCR and the sequencing of the target genomic regions. Table 1 lists the *M. luteus* strains used in this study.

### Olefin extraction

*Micrococcus luteus* and other *Micrococcales* cultures were grown at 30 °C to stationary phase and 10-mL samples were taken. The samples were centrifuged in glass vials with polytetrafluoroethylene (PTFE) screw caps. The cell pellets were resuspended in residual supernatant and 100 µL 100% acetic acid was added and thoroughly mixed. One milliliter of methanol and 4 mL hexane, amended with 10 or 50 µg/mL triacontane (Tokyo Chemical Industry) as an internal standard, were added. The mixture was shaken overnight at 640 rpm in a KS 130B shaker to extract the maximum amount of olefins possible. To facilitate phase separation, the samples were centrifuged and the upper hexane phase was used for analysis.

### Lipid and free fatty acid extraction

The cultures were grown to stationary phase and 2.5-mL samples were transferred to glass vials with PTFE screw caps. The cell suspension was thoroughly mixed with 100 µL 100% acetic acid, after which 500 µL 100 µg/mL eicosanoic acid (Larodan AB) solution in ethanol as an internal standard and 5 mL methanol/chloroform (1:1) were added. The mixture was shaken overnight at 640 rpm and after centrifugation, the lower chloroform phase was transferred to a new glass vial and completely evaporated. Lipid and free fatty acids were methylated by adding 500 µL 1.2 M hydrochloric acid in methanol and incubating at 50 °C overnight while shaking. The esterification reaction was quenched by adding 5 mL 100 mg/mL aqueous NaHCO_3_. Finally, the fatty acid methyl esters (FAMEs) were extracted with 1 mL hexane, and the hexane phase was transferred into a GC/MS vial for analysis.

### Gas chromatography/mass spectrometry

A QP2020 gas chromatograph/mass spectrometer (Shimadzu Corp.) was used to perform the GC/MS analysis. The gas chromatograph was equipped with a split or splitless injection system and a Rxi-5 ms (30 m × 0.25 mm i.d. × 0.25 µm) capillary column, and MS was operated under ionization by electron impact at 70 eV and 200 °C. The 1-µL samples were injected in the split or splitless mode. Helium flow was maintained at 1 mL/min. The temperature of the column and duration was 40 °C for 3 min, then increased to 280 °C at 20 °C/min, and finally held for 5 min for FAMEs or 8 min for olefins. Mass spectra were recorded at m/z (mass/charge) 45–500 at a rate of 0.2/s.

The extractable olefins were quantified by directly comparing their peak areas with that of triacontane (C30 alkane, 30 carbon atoms) of known concentration, which had been added to the hexane used to extract the olefins. FAMEs were quantified by directly comparing their peak areas with that of eicosanoic acid (C20:0) of known concentration, which had been added to the sample at the beginning of the extraction procedure. For comparison of the olefin amounts produced by different *Micrococcales* strains, the quantitative data were normalized to a culture density of OD_600_ = 1.

## Supplementary information

**Additional file 1: Figure S1.***Arthrobacter* strains. The total fatty acid and olefin chain lengths and isomer distribution, as well as absolute cellular amounts in complex medium. Values are the mean of at least two biological replicates. The error bars represent standard deviation. Except for *Arthrobacter oxydans*, which was < 15%, the standard deviations of the heat map values did not exceed 2%. Abbreviations: *isoiso*, *iso*-branched at both ends; *isoai*, *iso*-branched at one end and *anteiso*-branched at the other end; *aiai*, *anteiso*-branched at both ends; *isosc*, *iso*-branched at one end and no branching at the other end; *aisc*, *anteiso*-branched at one end and no branching at the other end; *scsc*, no branching at both ends; *even iso*, *iso*-branched even-numbered; *odd iso*, *iso*-branched odd-numbered; *ai*, *anteiso*-branched; *sc*, straight chain; *br*, branched; *br*-*un*, branched and unsaturated; *sc*-*un*, straight chain and unsaturated; OD, optical density [15].

**Additional file 2: Figure S2.***Brevibacterium/Curtobacterium* strains. The total fatty acid and olefin chain lengths and isomer distributions, as well as absolute cellular amounts in complex medium. The values are the mean of at least two biological replicates. The error bars represent standard deviation. Except for *Brevibacterium casei* (*Sc*hleifer, 492), which was < 6%, standard deviations of the heat map values did not exceed 3%. Notes: *homotypic synonyms of *Brevibacterium*. Abbreviations: *cyclo*, cyclic fatty acids; for other abbreviations see Additional file: 1 Figure S1 [15].

**Additional file 3: Figure S3.***Kocuria* strains. The total fatty acid and olefin chain lengths and isomer distributions, as well as absolute cellular amounts in complex medium. The values are the mean of at least two biological replicates. The error bars represent standard deviation. Except for *Kocuria sp.* MAW846M, which was < 6%, the standard deviations of the heat map values did not exceed 3%. Abbreviations: see Additional file: 1 Figure S1 [15].

**Additional file 4: Figure S4.***Micrococcus* strains. The total fatty acid and olefin chain lengths and isomer distributions, as well as absolute cellular amounts in complex medium. The values are the mean of at least two biological replicates. The error bars represent standard deviation. Except for *Micrococcus luteus* A31655 and *M. aurantiacus*, which were < 12%, the standard deviations of the heat map values did not exceed 5%. Abbreviations: see Additional file: 1 Figure S1 [15].

**Additional file 5: Figure S5.** Diverse *Micrococcales* strains. The total fatty acid and olefin chain lengths and isomer distributions, as well as absolute cellular amounts in complex medium. The values are the mean of at least two biological replicates. The error bars represent standard deviation. The standard deviations of the heat map values did not exceed 7%. Abbreviations: *cyclo*, cyclic fatty acids; for other abbreviations see Additional file: 1 Figure S1 [15].

## Data Availability

The datasets analyzed for generation of the phylogenetic tree during the current study are available on Github, [https://github.com/angelovangel/oleA-distribution-paper-dataset]. The residual data generated or analyzed during this study are included in this published article [and its supplementary information files].

## Abbreviations

*ai*-branched: *Anteiso*-branched
*aiai*-branched: *Anteiso*-branched at both ends
cis-configured: The main substituents of the double bond are orientated to the same side of the molecule
C20:0: 20 carbon atoms and no double bond
C24: 24 carbon atoms
C29: 29 carbon atoms
C30: 30 carbon atoms
FAME: Fatty acid methyl ester
GC/MS: Gas chromatography/mass spectrometry
high-GC: High guanine plus cytosine content of the genome
*isoiso*-branched: *Iso*-branched at both ends
LB: Lysogeny broth
m/z: Mass/charge
ole: Olefin biosynthesis pathway
olex: *OleA* or *oleABCD* exchange mutants
PCR: Polymerase chain reaction
SSN: Sequence similarity network
