# Supplementary material for: Distribution and diversity of olefins and olefin-biosynthesis genes in Gram-positive bacteria
Source: Biotechnol Biofuels. 2020 Apr 15;13:70. doi: 10.1186/s13068-020-01706-y (PMC7158056; doi:10.1186/s13068-020-01706-y)

olefins

fatty acids

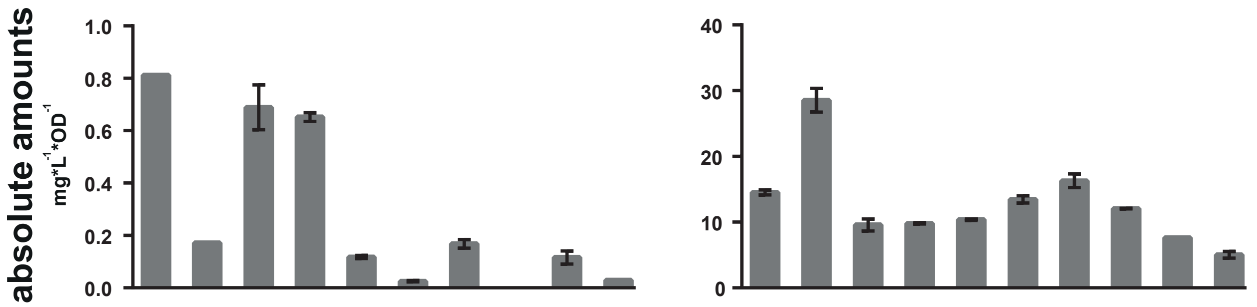

isomers (olefins)

	Arthrobacter agilis	Arthrobacter atrocyaneus	Arthrobacter aurescens	Arthrobacter citreus	Arthrobacter crystallopoites	Arthrobacter oxydans	Arthrobacter polychromogenes	Arthrobacter sp.	Arthrobacter sulfureus	Arthrobacter uratoxydans
isoiso	35.14	0	13.31	0	12.40	0	10.98	16.27	6.94	27.17
isoai	46.37	10.48	19.62	15.00	17.43	35.04	21.39	51.24	24.61	48.53
aiai	13.87	89.52	67.08	85.00	70.17	64.96	67.63	32.49	68.45	24.31
isosc	3.01	0	0	0	0	0	0	0	0	0
aisc	1.60	0	0	0	0	0	0	0	0	0
scsc	0	0	0	0	0	0	0	0	0	0

isomers (fatty acids)

	Arthrobacter agilis	Arthrobacter atrocyaneus	Arthrobacter aurescens	Arthrobacter citreus	Arthrobacter crystallopoites	Arthrobacter oxydans	Arthrobacter sp.	Arthrobacter polychromogenes	Arthrobacter sulfureus	Arthrobacter uratoxydans
iso even	17.38	6.14	4.14	2.51	7.17	5.18	9.05	7.74	5.60	5.96
iso odd	13.23	19.55	12.38	18.17	3.80	4.51	13.93	18.84	23.11	35.43
ai	64.87	73.60	82.41	76.47	88.09	89.46	68.89	67.08	70.09	57.07
sc	4.52	0.72	1.08	2.85	0.93	0.85	8.12	6.33	1.19	1.54
br-un	0	0	0	0	0	0	7.36	7.18	0.16	0
sc-un	0	0	0	0	0	0	4.25	3.35	0	0

chain lengths (olefins)

	Arthrobacter agilis	Arthrobacter atrocyaneus	Arthrobacter aurescens	Arthrobacter citreus	Arthrobacter crystallopoites	Arthrobacter oxydans	Arthrobacter polychromogenes	Arthrobacter sp.	Arthrobacter sulfureus	Arthrobacter uratoxydans
C20	0	0	0	0	0	0	0	0	0	0
C21	0	0	0	0	0	0	0	0	0	0
C22	0	0	0	0	0	0	0	0	0	0
C23	0	0	0	0	0	0	0	0	0	0
C24	0	0	0	0	0	0	0	0	0	0
C25	0	0	0	0	0	0	0	0	0	0
C26	0	0	0	0	0	0	0	0	0	0
C27	20.00	0	0	0	0	0	0	0	0	0
C28	18.96	0	0	0	0	0	0	10.02	0	0
C29	56.59	100.00	100.00	100.00	100.00	100.00	100.00	89.98	100.00	100.00
C30	4.45	0	0	0	0	0	0	0	0	0
C31	0	0	0	0	0	0	0	0	0	0

chain lengths (fatty acids)

	Arthrobacter agilis	Arthrobacter atrocyaneus	Arthrobacter aurescens	Arthrobacter citreus	Arthrobacter crystallopoites	Arthrobacter oxydans	Arthrobacter sp.	Arthrobacter polychromogenes	Arthrobacter sulfureus	Arthrobacter uratoxydans
C11	0	0	0	0	0	0	0	0	0	0
C12	0	0	0	0	0	0	0	0.06	0	0
C13	0.02	0.03	0	0	0	0	0	0	0	0
C14	1.81	0.63	0.54	0.91	0.64	0.79	1.62	1.30	1.04	0.83
C15	64.66	65.02	79.56	88.45	67.89	77.21	58.98	63.39	85.09	67.41
C16	19.65	6.16	4.56	3.39	7.34	5.16	15.39	11.06	5.59	6.34
C17	13.41	28.09	15.22	6.29	24.01	16.76	23.84	22.85	8.12	25.22
C18	0.44	0.07	0.12	0.96	0.12	0.07	0.17	1.35	0.16	0.19
C19	0	0	0	0	0	0	0	0	0	0
C20	0	0	0	0	0	0	0	0	0	0

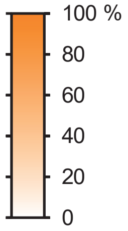

Supplement: Supplementary file 1 — Additional file 1: Figure S1.Arthrobacter strains. The total fatty acid and olefin chain lengths and isomer distribution, as well as absolute cellular amounts in complex medium. Values are the mean of at least two biological replicates. The error bars represent standard deviation. Except for Arthrobacter oxydans, which was < 15%, the standard deviations of the heat map values did not exceed 2%. Abbreviations: isoiso, iso-branched at both ends; isoai, iso-branched at one end and anteiso-branched at the other end; aiai, anteiso-branched at both ends; isosc, iso-branched at one end and no branching at the other end; aisc, anteiso-branched at one end and no branching at the other end; scsc, no branching at both ends; even iso, iso-branched even-numbered; odd iso, iso-branched odd-numbered; ai, anteiso-branched; sc, straight chain; br, branched; br-un, branched and unsaturated; sc-un, straight chain and unsaturated; OD, optical density [15]. [file 13068_2020_1706_MOESM1_ESM.pdf]
