# Supplementary material for: Distribution and diversity of olefins and olefin-biosynthesis genes in Gram-positive bacteria
Source: Biotechnol Biofuels. 2020 Apr 15;13:70. doi: 10.1186/s13068-020-01706-y (PMC7158056; doi:10.1186/s13068-020-01706-y)
Supplement: Supplementary file 2 — Additional file 2: Figure S2.Brevibacterium/Curtobacterium strains. The total fatty acid and olefin chain lengths and isomer distributions, as well as absolute cellular amounts in complex medium. The values are the mean of at least two biological replicates. The error bars represent standard deviation. Except for Brevibacterium casei (Schleifer, 492), which was < 6%, standard deviations of the heat map values did not exceed 3%. Notes: *homotypic synonyms of Brevibacterium. Abbreviations: cyclo, cyclic fatty acids; for other abbreviations see Additional file: 1 Figure S1 [15]. [file 13068_2020_1706_MOESM2_ESM.pdf]

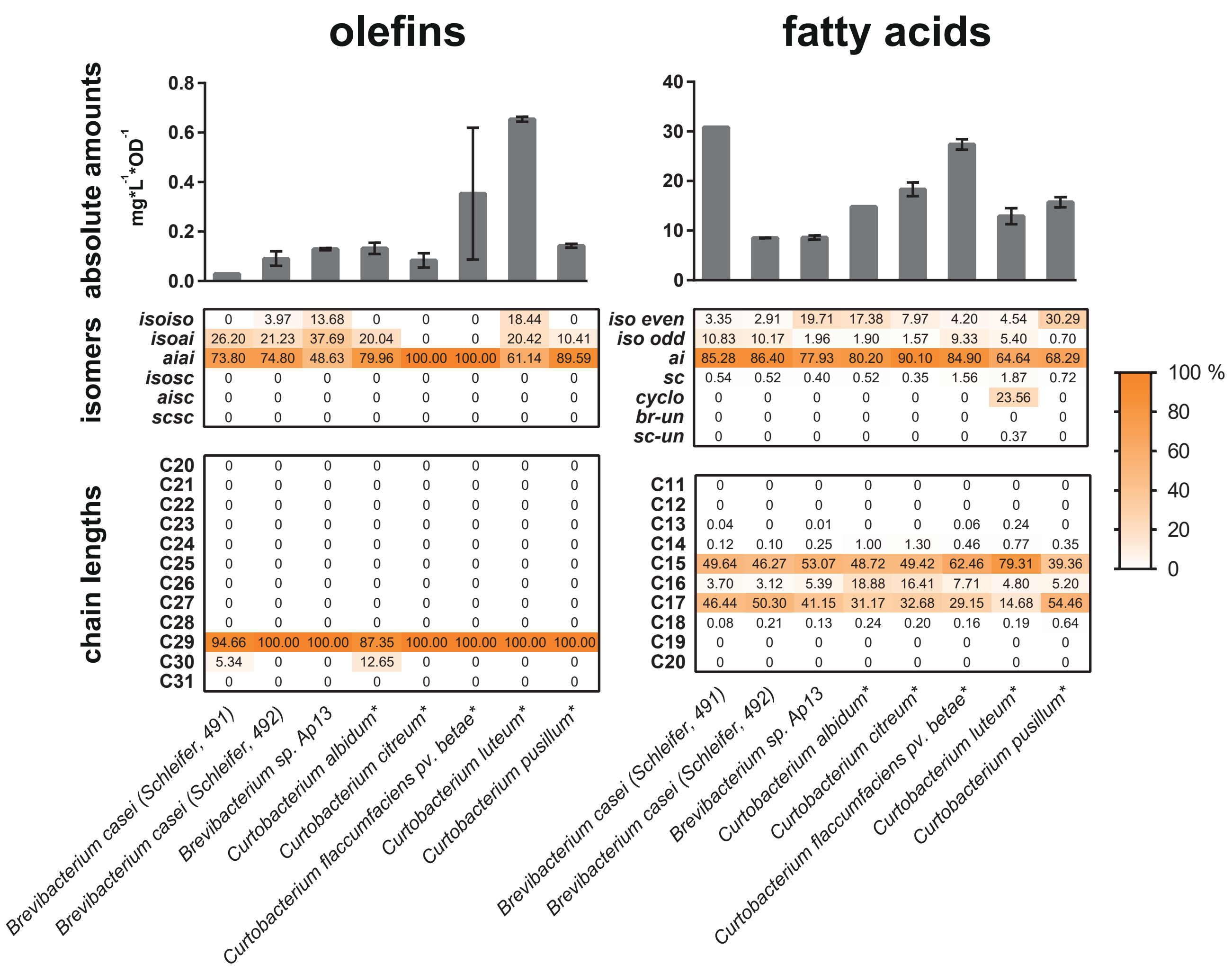
olefins
fatty acids
absolute amounts
mg*L-1*OD-1
0.8
0.6
0.4
0.2
0.0
40
30
20
10
0
isomers
isoiso 0 3.97 13.68 0 0 0 18.44 0
isoai 26.20 21.23 37.69 20.04 0 0 20.42 10.41
aiai 73.80 74.80 48.63 79.96 100.00 100.00 61.14 89.59
isosc 0 0 0 0 0 0 0 0
aisc 0 0 0 0 0 0 0 0
scsc 0 0 0 0 0 0 0 0
iso even 3.35 2.91 19.71 17.38 7.97 4.20 4.54 30.29
iso odd 10.83 10.17 1.96 1.90 1.57 9.33 5.40 0.70
ai 85.28 86.40 77.93 80.20 90.10 84.90 64.64 68.29
sc 0.54 0.52 0.40 0.52 0.35 1.56 1.87 0.72
cyclo 0 0 0 0 0 0 23.56 0
br-un 0 0 0 0 0 0 0 0
sc-un 0 0 0 0 0 0 0.37 0
100 %
80
60
40
20
0
chain lengths
C20 0 0 0 0 0 0 0 0
C21 0 0 0 0 0 0 0 0
C22 0 0 0 0 0 0 0 0
C23 0 0 0 0 0 0 0 0
C24 0 0 0 0 0 0 0 0
C25 0 0 0 0 0 0 0 0
C26 0 0 0 0 0 0 0 0
C27 0 0 0 0 0 0 0 0
C28 0 0 0 0 0 0 0 0
C29 94.66 100.00 100.00 87.35 100.00 100.00 100.00 100.00
C30 5.34 0 0 12.65 0 0 0 0
C31 0 0 0 0 0 0 0 0
C11 0 0 0 0 0 0 0 0
C12 0 0 0 0 0 0 0 0
C13 0.04 0 0.01 0 0 0.06 0.24 0
C14 0.12 0.10 0.25 1.00 1.30 0.46 0.77 0.35
C15 49.64 46.27 53.07 48.72 49.42 62.46 79.31 39.36
C16 3.70 3.12 5.39 18.88 16.41 7.71 4.80 5.20
C17 46.44 50.30 41.15 31.17 32.68 29.15 14.68 54.46
C18 0.08 0.21 0.13 0.24 0.20 0.16 0.19 0.64
C19 0 0 0 0 0 0 0 0
C20 0 0 0 0 0 0 0 0
Brevibacterium casei (Schleifer, 491)
Brevibacterium casei (Schleifer, 492)
Brevibacterium sp. Ap13
Curtobacterium albidum*
Curtobacterium citreum*
Curtobacterium flaccumfaciens pv. betae*
Curtobacterium luteum*
Curtobacterium pusillum*
