# Supplementary material for: Distribution and diversity of olefins and olefin-biosynthesis genes in Gram-positive bacteria
Source: Biotechnol Biofuels. 2020 Apr 15;13:70. doi: 10.1186/s13068-020-01706-y (PMC7158056; doi:10.1186/s13068-020-01706-y)

olefins

absolute amounts (mg*L^{-1}*OD^{-1})

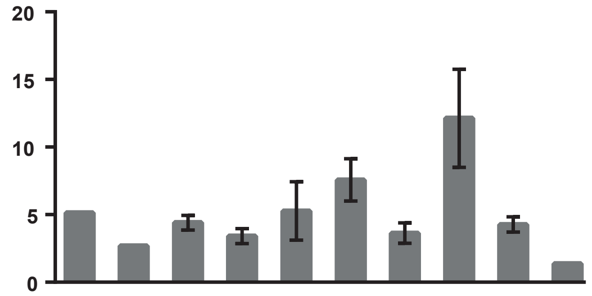

isomers

	K. rosea (Liebl)	K. rosea (Schleifer)	Kocuria palustris	Kocuria palustris DSM 11925	Kocuria palustris DSM 20319	Kocuria palustris DSM 20320	Kocuria sp. 3312	Kocuria sp. 3352	Kocuria sp. MAW846M	Kocuria sp. AT3343
isoiso	19.77	17.88	26.48	11.33	20.52	28.59	63.51	58.93	53.89	23.97
isoai	34.94	30.80	31.19	22.62	11.20	15.25	6.81	22.76	13.94	39.56
aiai	24.21	34.35	9.45	14.62	6.16	4.61	2.77	15.12	1.87	27.24
isosc	7.33	6.10	11.27	5.56	38.16	35.47	8.80	1.84	10.56	4.17
aisc	11.83	10.88	9.68	7.09	1.98	3.61	5.71	1.35	3.27	5.06
scsc	1.12	0	10.97	24.24	21.91	11.68	12.40	0	14.28	0

chain lengths

	K. rosea (Liebl)	K. rosea (Schleifer)	Kocuria palustris	Kocuria palustris DSM 11925	Kocuria palustris DSM 20319	Kocuria palustris DSM 20320	Kocuria sp. 3312	Kocuria sp. 3352	Kocuria sp. MAW846M	Kocuria sp. AT3343
C20	0	0	0	3.99	0	0	2.00	0	0	0
C21	0	0	7.71	6.53	5.01	4.54	2.91	0.27	1.82	0
C22	0.81	1.10	8.47	7.86	6.61	8.63	3.93	0.32	4.12	0
C23	6.86	3.92	44.12	47.41	46.84	41.98	30.72	9.76	26.93	3.10
C24	19.65	16.51	32.02	25.47	29.95	35.09	19.49	11.60	30.47	10.21
C25	66.35	72.46	6.91	8.75	8.99	7.62	40.08	63.56	32.87	82.44
C26	4.92	3.87	0.76	0	1.34	0.98	0.86	4.00	1.77	0.58
C27	1.42	2.14	0	0	0.20	0	0	9.88	0.94	3.10
C28	0	0	0	0	0	0	0	0.26	0.10	0.57
C29	0	0	0	0	0.99	0.37	0	0.36	0.61	0
C30	0	0	0	0	0	0	0	0	0	0
C31	0	0	0	0	0	0	0	0	0	0

fatty acids

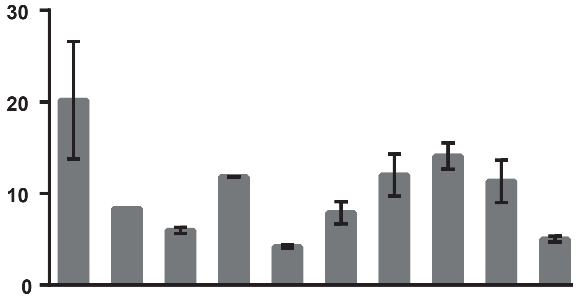

isomers

	K. rosea (Liebl)	K. rosea (Schleifer)	Kocuria palustris	Kocuria palustris DSM 11925	Kocuria palustris DSM 20319	Kocuria palustris DSM20320	Kocuria sp. 3312	Kocuria sp. 3352	Kocuria sp. MAW846M	Kocuria sp. AT3343
iso even	1.75	3.62	4.53	9.65	8.34	6.08	10.42	11.69	10.65	1.89
iso odd	22.52	19.66	6.59	8.22	8.94	4.85	17.76	29.13	10.62	25.25
ai	72.13	71.16	86.28	79.86	78.01	84.76	61.62	56.31	69.05	62.66
sc	3.60	5.55	2.60	2.28	4.71	4.31	10.21	2.87	18.20	0
br-un	0	1.43	0	0	0	0	0	0	0	10.20
sc-un	0	2.86	0	0	0	0	0	0	0	1.68

chain lengths

	K. rosea (Liebl)	K. rosea (Schleifer)	Kocuria palustris	Kocuria palustris DSM 11925	Kocuria palustris DSM 20319	Kocuria palustris DSM20320	Kocuria sp. 3312	Kocuria sp. 3352	Kocuria sp. MAW846M	Kocuria sp. AT3343
C11	0	0	0	0	0	0	0	0	0	0
C12	0	0	0	0	0	0	0	0	0	0
C13	0	0	0	1.74	0	0.41	1.59	3.86	1.56	0.84
C14	3.77	4.11	2.71	7.89	4.37	3.71	8.76	4.58	7.21	2.52
C15	93.06	88.59	86.28	78.34	78.01	84.70	72.48	64.96	70.52	87.54
C16	1.29	4.93	4.42	4.04	8.68	6.68	10.79	9.38	12.12	4.74
C17	1.89	2.36	6.59	7.99	8.94	4.50	6.37	17.21	8.59	1.74
C18	0	0	0	0	0	0	0	0	0	2.62
C19	0	0	0	0	0	0	0	0	0	0
C20	0	0	0	0	0	0	0	0	0	0

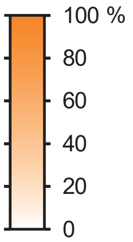

Supplement: Supplementary file 3 — Additional file 3: Figure S3.Kocuria strains. The total fatty acid and olefin chain lengths and isomer distributions, as well as absolute cellular amounts in complex medium. The values are the mean of at least two biological replicates. The error bars represent standard deviation. Except for Kocuria sp. MAW846M, which was < 6%, the standard deviations of the heat map values did not exceed 3%. Abbreviations: see Additional file: 1 Figure S1 [15]. [file 13068_2020_1706_MOESM3_ESM.pdf]
