# Supplementary figures and images for: Distribution and diversity of olefins and olefin-biosynthesis genes in Gram-positive bacteria
Source: Biotechnol Biofuels. 2020 Apr 15;13:70. doi: 10.1186/s13068-020-01706-y (PMC7158056; doi:10.1186/s13068-020-01706-y)

## olefins

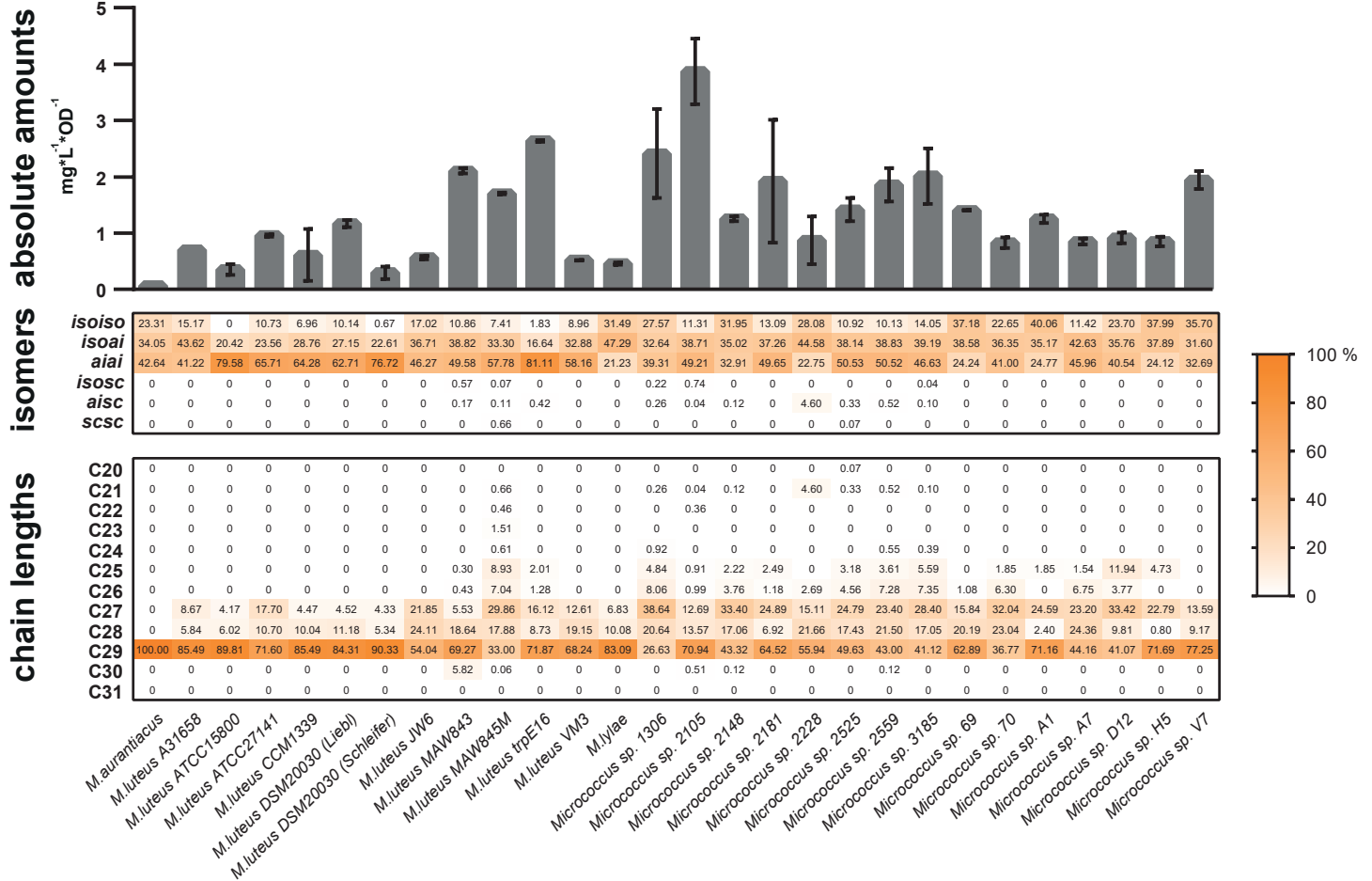

## fatty acids

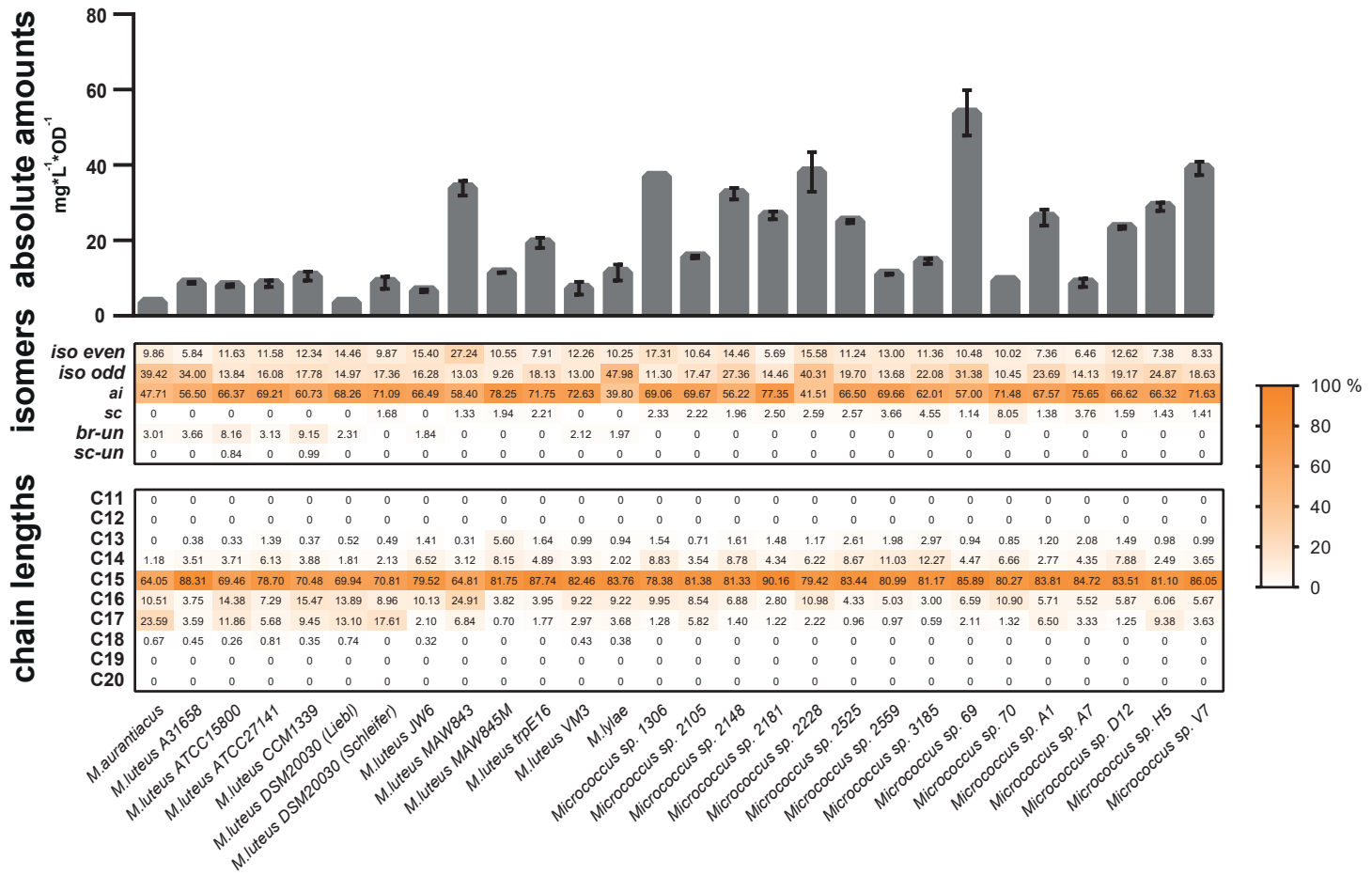

Supplement: Supplementary file 4 — Additional file 4: Figure S4.Micrococcus strains. The total fatty acid and olefin chain lengths and isomer distributions, as well as absolute cellular amounts in complex medium. The values are the mean of at least two biological replicates. The error bars represent standard deviation. Except for Micrococcus luteus A31655 and M. aurantiacus, which were < 12%, the standard deviations of the heat map values did not exceed 5%. Abbreviations: see Additional file: 1 Figure S1 [15]. [file 13068_2020_1706_MOESM4_ESM.pdf]
