# Supplementary material for: Distribution and diversity of olefins and olefin-biosynthesis genes in Gram-positive bacteria
Source: Biotechnol Biofuels. 2020 Apr 15;13:70. doi: 10.1186/s13068-020-01706-y (PMC7158056; doi:10.1186/s13068-020-01706-y)
Supplement: Supplementary file 5 — Additional file 5: Figure S5. Diverse Micrococcales strains. The total fatty acid and olefin chain lengths and isomer distributions, as well as absolute cellular amounts in complex medium. The values are the mean of at least two biological replicates. The error bars represent standard deviation. The standard deviations of the heat map values did not exceed 7%. Abbreviations: cyclo, cyclic fatty acids; for other abbreviations see Additional file: 1 Figure S1 [15]. [file 13068_2020_1706_MOESM5_ESM.pdf]

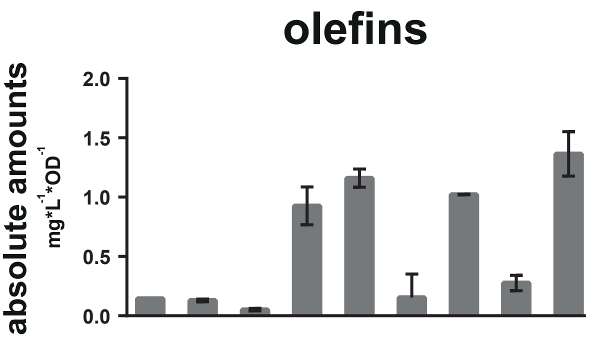

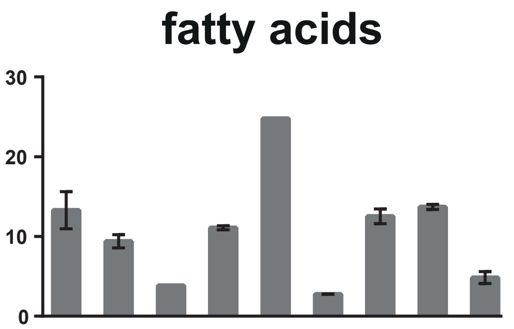

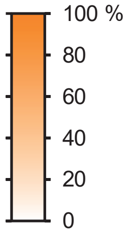

olefins – isomers

	Clavibacter michiganensis subsp. *nebraskensis*	*Glutamicibacter nicotinae*	*Glutamicibacter protophormiae*	*Kytococcus sedentarius* TW93	*Microbacterium imperiale*	*Dermacoccus* sp. blue	*Paenarthrobacter ilicis*	*Pseudarthrobacter chlorophenolicus*	*Rothia* sp.
isoiso	7.55	0	16.78	25.71	0	2.15	1.34	18.95	1.47
isoai	33.01	18.03	47.43	4.98	14.23	49.67	6.73	31.71	12.52
aiai	59.44	81.97	35.79	10.27	85.77	48.18	91.93	49.34	86.00
isosc	0	0	0	33.87	0	0	0	0	0
aisc	0	0	0	3.74	0	0	0	0	0
scsc	0	0	0	18.16	0	0	0	0	0

olefins – chain lengths

	Clavibacter michiganensis subsp. *nebraskensis*	*Glutamicibacter nicotinae*	*Glutamicibacter protophormiae*	*Kytococcus sedentarius* TW93	*Microbacterium imperiale*	*Dermacoccus* sp. blue	*Paenarthrobacter ilicis*	*Pseudarthrobacter chlorophenolicus*	*Rothia* sp.
C20	0	0	0	0	0	0	0	0	0
C21	0	0	0	2.98	0	0	0	0	0
C22	0	0	0	10.51	0	0	0	0	0
C23	0	0	0	27.65	0	0	0	0	0
C24	0	0	0	19.35	0	0	0	0	0
C25	0	0	0	20.30	0	0	0	0	0
C26	0	0	0	0.09	0	0	0	0	0
C27	12.73	0	0	3.11	13.42	11.83	0.67	2.81	3.58
C28	1.24	0	0	1.59	6.52	5.17	0.75	6.86	11.33
C29	86.03	100.00	100.00	14.14	76.40	80.70	95.80	90.33	85.09
C30	0	0	0	0	3.66	0.55	1.40	0	0
C31	0	0	0	0	0	1.74	1.39	0	0

fatty acids – isomers

	Clavibacter michiganensis subsp. *nebraskensis*	*Glutamicibacter nicotinae*	*Glutamicibacter protophormiae*	*Kytococcus sedentarius* TW93	*Microbacterium imperiale*	*Dermacoccus* sp. blue	*Paenarthrobacter ilicis*	*Pseudarthrobacter chlorophenolicus*	*Rothia* sp.
iso even	7.37	17.51	7.36	7.83	4.54	36.22	5.08	11.41	12.86
iso odd	14.95	9.46	33.41	39.39	5.40	33.14	2.87	17.09	0.43
ai	72.05	65.87	55.44	41.56	64.64	2.03	91.05	65.63	84.79
sc	5.62	7.17	3.79	17.00	1.87	8.53	1.00	5.87	0
cyclo	0	0	0	0	23.56	0	0	0	0
br-un	0.32	0	0	0	0	20.67	0	0	1.93
sc-un	0	0	0	0	0.37	10.78	0	0	0

fatty acids – chain lengths

	Clavibacter michiganensis subsp. *nebraskensis*	*Glutamicibacter nicotinae*	*Glutamicibacter protophormiae*	*Kytococcus sedentarius* TW93	*Microbacterium imperiale*	*Dermacoccus* sp. blue	*Paenarthrobacter ilicis*	*Pseudarthrobacter chlorophenolicus*	*Rothia* sp.
C11	0.24	0	0	0	0	0	0	4.71	0
C12	0.02	0	0	0	0	0	0	0	0
C13	0.80	0	0	1.51	0.02	0	0	0.09	0.38
C14	1.14	3.44	1.62	3.00	0.89	0	0.60	1.84	2.74
C15	61.38	58.10	68.06	51.09	40.54	10.69	74.63	61.12	78.86
C16	9.13	21.01	8.97	11.03	29.95	41.60	5.37	14.97	11.22
C17	27.04	17.32	21.08	33.37	28.43	37.74	19.29	16.95	6.28
C18	0.14	0.13	0.27	0	0.18	9.97	0.12	0.25	0.52
C19	0	0	0	0	0	0	0	0	0
C20	0	0	0	0	0	0	0	0	0
